# Comparison of the Diet Photograph Record to Weighed Dietary Record and 24 h Dietary Recall for Estimating Energy and Nutrient Intakes Among Chinese Preschoolers

**DOI:** 10.3389/fnut.2021.755683

**Published:** 2021-11-11

**Authors:** Yujie Xu, Ruonan Duan, Ping Feng, Wanke Gao, Dong Xing, Guo Cheng

**Affiliations:** ^1^Department of Nutrition, Food Safety, and Toxicology, West China School of Public Health and West China Fourth Hospital, Sichuan University, Chengdu, China; ^2^Shanxi Bethune Hospital, Shanxi Academy of Medical Sciences, Tongji Shanxi Hospital, Third Hospital of Shanxi Medical University, Taiyuan, China; ^3^Tongji Hospital, Tongji Medical College, Huazhong University of Science and Technology, Wuhan, China; ^4^Chongqing Municipal Center for Disease Control and Prevention, Chongqing, China; ^5^Laboratory of Molecular Translational Medicine, Center for Translational Medicine, Key Laboratory of Birth Defects and Related Diseases of Women and Children (Sichuan University), Ministry of Education, Department of Pediatrics, West China Second University Hospital, Sichuan University, Chengdu, China

**Keywords:** diet photograph record, weighed dietary record, 24 h dietary recall, dietary intake, validation, preschoolers

## Abstract

This study aimed to assess the relative validity of the diet photograph record (DP) for measuring the energy and nutrient intakes against the weighed dietary record (WD) and the 24 h dietary recall (HR) in the Chinese preschoolers. In this study, 40 preschool children aged 4–6 years and their parents were recruited from a kindergarten in southwest China. Dietary intake of the preschoolers on a same day, as estimated by the DP and the HR were compared with the WD. These three methods were administered by the three group of investigators independently. The mean differences, correlation coefficients, cross-classifications, and weighted κ, as well as the Bland–Altman plots were performed to assess the differences and agreements among the estimates from the DP, the HR, and the WD. For the DP and the HR, the estimates of energy and nutrient intakes were moderate to high correlated with the WD, with the higher coefficients ranging from 0.73 to 0.94 for the DP. Both the methods tended to underestimate the dietary intake, but the differences from the known weights using the DP were significantly smaller than those using the HR. The weighed κ values ranking the preschoolers ranged from 0.48 to 0.80 for the DP and ranged from 0.28 to 0.64 for the HR. Furthermore, the Bland–Altman plots indicated a better agreement between the DP and the WD for estimating energy and nutrient intakes. This DP is a valid tool for measuring energy and nutrient intakes among the preschoolers.

## Introduction

Proper energy and nutrient intake in the preschool children are crucial to maintain growth and development. An accurate assessment of the dietary data in this population is challenging, making it difficult to understand whether their diets are adequate (1–3). The dietary assessment methods that are used to assess the energy and nutrient intakes of preschoolers include weighed dietary record (WD) (4), direct diet observation (5), food frequency questionnaires (6), food records (6), and dietary recalls (6). There are well-known limitations to each method. Direct observation is an accurate method for estimating the dietary intakes in public eating situations but is disruptive of the regular eating environment (7, 8). The self-reported methods, e.g., the food frequency questionnaires, food records, and dietary recalls are questioned for the accuracy of memory or portion size estimation (9–11). In children aged 0.5–6 years, the WD is reported to provide the most accurate dietary estimates (4). However, it is time-consuming and costly (12), and may be impractical for the preschoolers who have multiple eating occasions in different settings outside of the home.

Progress in technology has advanced the development of innovative ways to overcome the current limitations in estimating dietary intakes (13, 14). A digital diet photography record (DP), which aims to capture all the eating occasions with food images, provides an opportunity to minimize the errors from the self-report estimates (15). This image-based instrument offers a lower respondent burden and provides valid dietary estimates. Williamson et al. (16) reported that the energy and nutrient estimates from the DP associated highly with weighed estimates (*r* > 0.90) with minimal mean differences (<6 g). Agreement among the human raters who evaluate dietary intakes using the DP was also high (17). Currently, the DP is validated for measuring food intakes of the preschool children, adolescents, and adults in the free-living and laboratory conditions (17–22). However, the feasibility and accuracy of this method with the preschool children is only done for a single meal not total daily intake (21, 23, 24). Also, these initial studies cover a small number of food groups (with a maximum 24 kinds of food items) and conduct in a controlled eating environment, not free-living conditions (21, 23).

Measuring the daily energy and nutrient intakes of the Chinese preschoolers in a kindergarten, we aimed to test the relative validity of the DP in comparison with the WD and to compare the estimates of dietary intakes using the DP with the estimates using a well-established method, i.e., the 24 h dietary recall (HR).

## Methods

### Study Design

In this study, the WD was the criterion. For the validation of the HR and the DP, 40 preschool children aged 4–6 years and their parents from a kindergarten in Chengdu, Sichuan, southwest China, were enrolled in March 2019. The convenience sampling was used to recruit the potential candidates from the First kindergarten of Sichuan University in Sichuan, China. To be eligible for participation, the preschoolers were required to meet the following criteria: no food-related allergy (e.g., lactose intolerance), metabolic diseases (e.g., type I diabetes), and current gastrointestinal problems (e.g., diarrhea and constipation) that could impair their ability to keep a regular diet and had a caregiver who volunteered to join and cooperate with the research staff in the study procedure. This study was approved by the Ethics Committee of Sichuan University and the written consent from the parents of the preschool children and verbal assent from the preschoolers were also obtained.

The preschoolers were observed for a 24-h period and received 4–5 meals, i.e., breakfast, lunch, dinner, and morning/afternoon snacks. Food for regular meals (breakfast, lunch, and dinner) was provided by the kindergarten according to their usual menu and timetable, while the morning and afternoon snacks were brought from the home of every child based on their preferences. Since children tend to share their snacks, we removed the possible bias by not allowing children to share. For the evaluation of food intake, the DP and the WD were performed on the same day, and the HR was performed on the next day, which were conducted by the three group of investigators who were completely independent from each other: (1) four investigators in the DP group needed to complete training on parents and estimation on food images; (2) two research staff of the HR group used face-to-face interviews to collect dietary data; (3) six investigators in the WD group were responsible for weighing raw weight and final cooked weight of each dish, as well as all foods consumed by each preschooler. Before the investigation phase, all the parents received training on the instructions for how the DP should be completed and practiced using the smartphone to capture and transmit the image of foods consumed by their child. The materials necessary for completing the DP included grid background paper, standard tableware (two plates, two bowls, and one cup), and mini program two-dimensional code was given to all the participants.

### DP—Test Method 1

**Figure 1:**
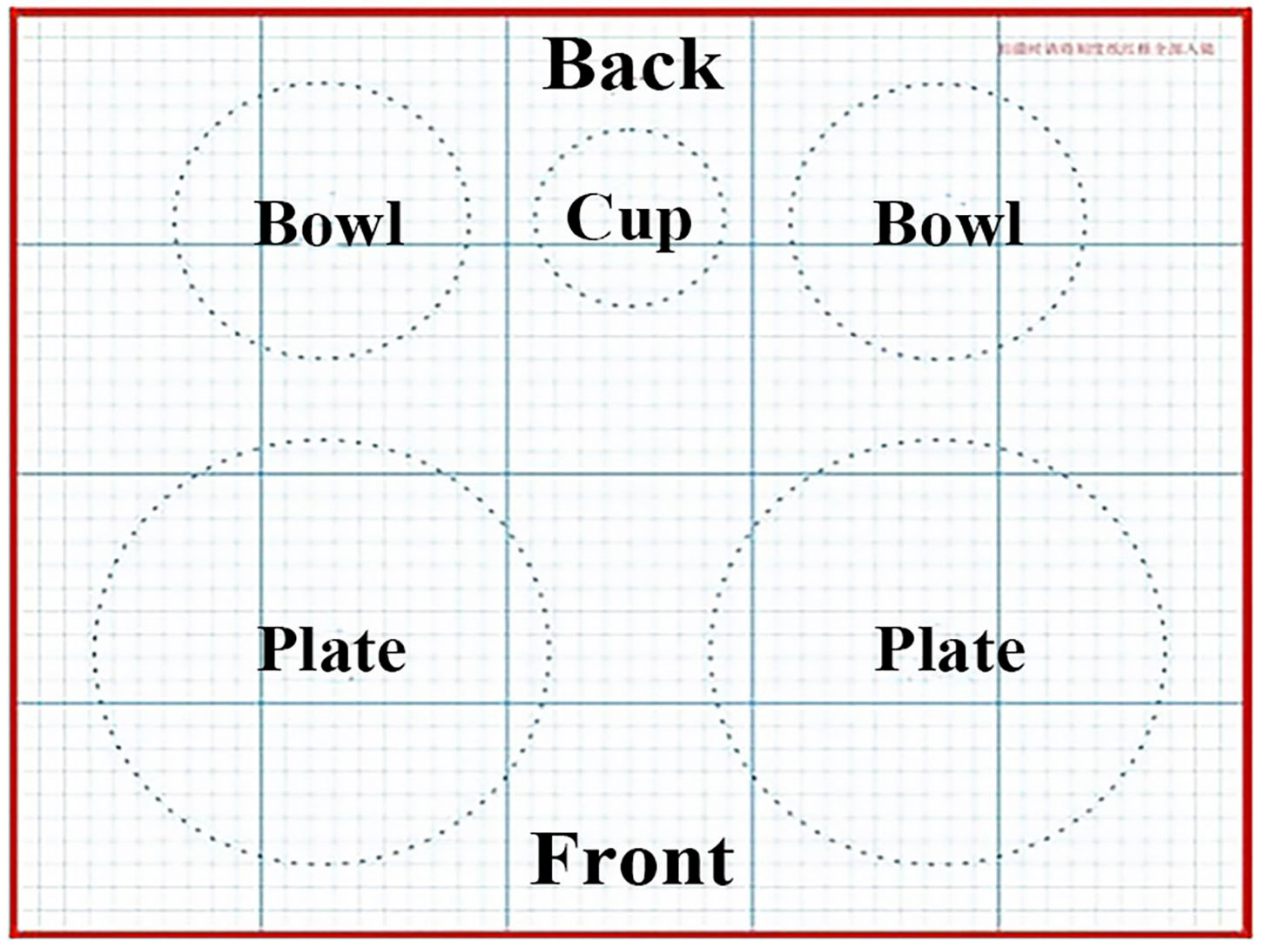
The grid background paper necessary for the diet photography record (DP). The upper smaller circles were the position of two bowls and a cup, and the lower were the position of two plates.

**Figure 2:**
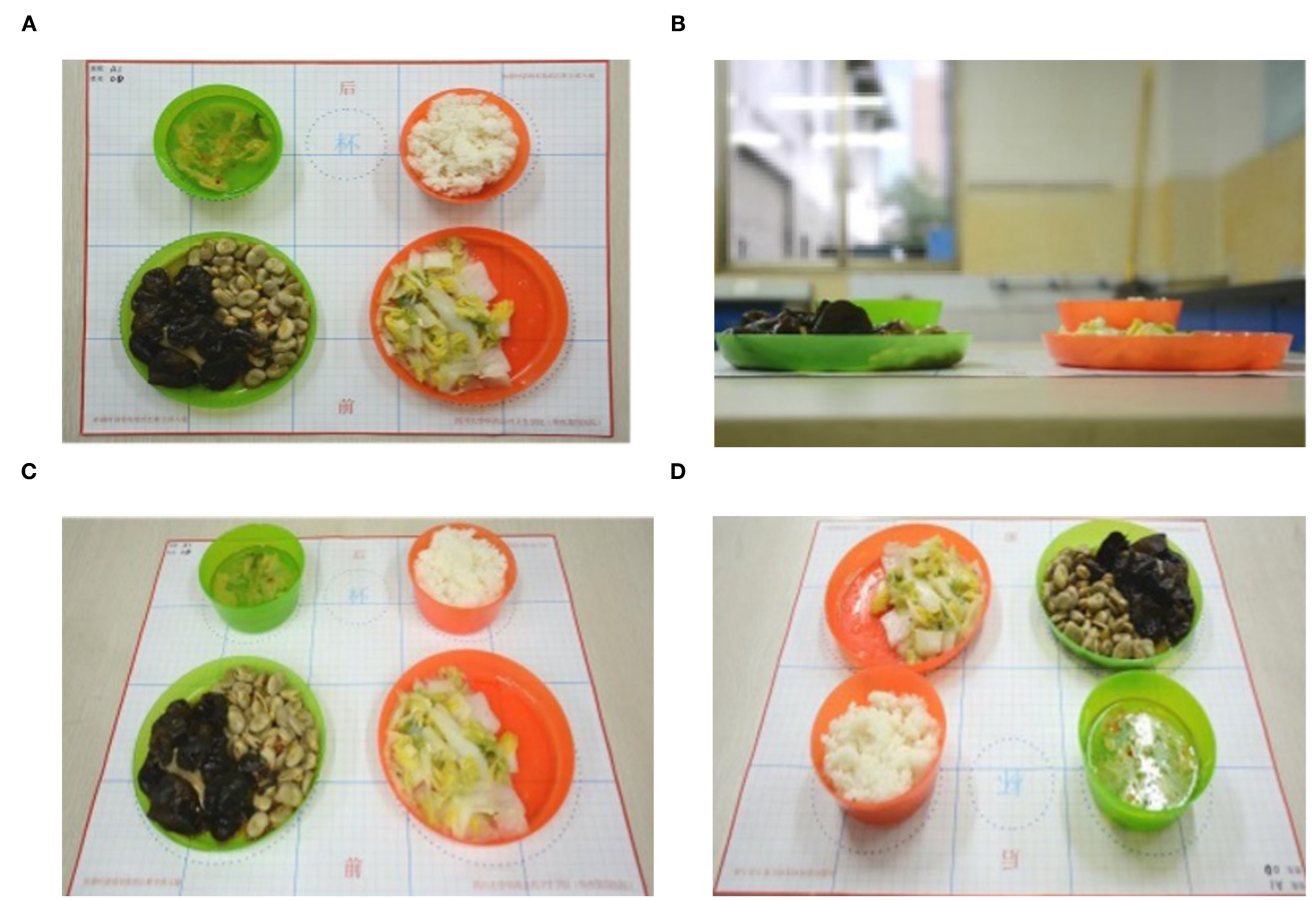
An example of the standard food photographs from the four directions. **(A)** The top, **(B)** the side, **(C)** the 45-degree angle above the forward, and **(D)** the 45-degree angle above the back.

The parents were asked to put and spread out the foods and beverage that were consumed into standard tableware, then, locate them on the grid background paper, where the position of plates, bowls and cup had been delineated (Figure 1). For each meal, the parents used their smartphone to take before and after pictures of all the foods consumed by each preschooler and any additional servings, except for water. The photographs of total foods before eating episode and corresponding plate waste after eating episode were to be taken from the four directions, i.e., the top, the side, the 45-degree angle above the forward, and the 45-degree angle above the back, as well as with proper lighting and an arm-length distance between the camera and the plate of each preschooler (Figure 2, standard example). After each meal, the parents were required to log in to “Dietary Assistant” to upload their images, which were transmitted in real time to the server and stored. The “Dietary Assistant” was designed by our research group in a form of online platform, using the WeChat application (a widely used social media among the Chinese developed by the Tencent Company in Shenzhen, China) as a carrier. The parents could obtain it through searching by the name or scanning by the mini program two-dimensional code in the WeChat.

To guarantee the accuracy of the DP gram estimates as compared with the actual gram, a standard food photograph library that consisted of an archive of more than 1,000 photographs of common foods consumed by the Chinese preschoolers and each with different gram of the same food was created. In most of these photographs, the food items, i.e., vegetables and meat, were mixed together based on the standard household cooking procedures, such as pan-frying, deep-frying, boiling, steaming, and roasting. The food images of dishes that were made of single foods were also available in our library. Three human raters with nutritional background had an educational training in how to use the DP application for gram weight estimation process. Each rater then practiced digital photography estimation of foods using the software and sample photographs. The estimations of each rater needed to fall within 20% of the actual values for each food item to be considered acceptable. During training, the raters practiced on a minimum of 120 images, with the intraclass correlation coefficients of more than 0.85, which indicated a good reliability among these raters.

The food intakes were estimated using the data exported from the “Dietary Assistant” application, and the photographs were checked for accuracy and completeness in real time. Two raters individually estimated the gram value of each food item in the “before” photograph by comparing their food images to the images of foods with a known weight. The “before” photograph was matched to a proper food group: (i) grains, (ii) legumes, (iii) vegetables, (iv) fruits, (v) meat, (vi) poultry, (vii) milk and dairy products, (viii) eggs, (ix) sea foods, and (x) snack foods. To estimate plate waste, the same procedure was followed using the “after” photograph. When the estimates of two raters of the same food differed by ≥30%, the estimation by a third rater would be conducted. The conflicts in rater estimates were discussed as a group to arrive at a consensus. The estimates of the rater were entered into a formed excel sheet, gram value of food intakes was calculated by subtracting the plate waste estimate from the food estimate.

### HR—Test Method 2

The HR recording the food consumption of the participants was collected by the research staff in face-to-face interviews on the day after conducting the DP and the WD. All the investigators were trained, e.g., in how to estimate the portion size and questioning skills with neutral attitudes. The parents were asked to recall the amount of all foods and beverages consumed by their child and corresponding timing, as well as a series of questions for the type of food items and cooking methods. For accurate estimation of serving sizes, standard tableware, commonly used in Chinese household, was provided for helping the participants determine their intake. Then, the quantities of foods and beverages recorded in the HR were converted from portion sizes into grams.

### WD—Reference Method

As the reference method, the WD was completed on the same day of the DP. A total of four research staffs weighed and recorded the raw weight of food before cooking, as well as the final cooked weight to obtain the cooking loss, which was determined by the ratio of difference between the raw weight and final cooked weight. The calculation was as follows: cooking loss (%) =100 × (raw weight – final cooked weight)/raw weight. All the foods and beverages consumed by the preschoolers should be weighed by the two research staffs to the nearest 0.1 g with the help of an electronic food scale (Thomas SWT3K01, Shanghai, China). Plate waste was also weighed and documented by the research staff to allow total intake estimates by difference (amount served minus plate waste). Moreover, the cooking loss ratio was used for transforming into raw weight of each food.

### Data Evaluation

The individual mean intakes of energy and 15 nutrients (protein, total fat, carbohydrate, vitamin A, B1, B2, C, E, potassium, sodium, calcium, magnesium, iron, zinc, and selenium) recorded in the DP, HR, and WD were converted from the food items using the continuously updated in-house nutrient database based on the NCCW software (version 11.0, 2014; Qingdao University Medical College, Shandong, China). The NCCW contains information on energy and 36 nutrients for >1,527 entries (944 basic food items, 562 food products, and 21 dietary supplements), which reflects the China Food Composition (25).

### Statistical Analysis

All the analyses were performed using the SAS statistical software package (SAS, version 9.4, SAS Institute Inc., Cary, NC, USA). The value of *P* < 0.05 was considered as a significance level, except for the interaction, where *P* < 0.01 was statistically significant. Since the food choice and eating behavior varies between sex, and sex has been reported to contribute partly to the variance in underreporting error of self-report dietary recall (26), we tested for the interactions with sex. No interaction with sex was detected through using the linear regression models, thus we pooled data from girls and boys for all the tests. Since most data of nutrient intakes were not normally distributed, the non-parametric methods were performed to evaluate the validity of the DP or the HR compared with the WD. The values in the results and tables were presented as medians (Q1, Q3).

The mean differences in weight of the food groups, intakes of energy, and nutrient between recorded in the DP, the HR, and the WD were all calculated. The significance of the differences was tested using Wilcoxon's signed-rank test. To assess the associations among the food group, energy, and nutrient intakes obtained by the three methods, the Spearman's rank correlation coefficients were evaluated. The Bonferroni correction was also applied to minimize the probability Type I error. Additionally, the preschoolers were grouped into quartiles for energy and each nutrient intake to test the agreement in classifying the participants according to their dietary intakes, as estimated by the three methods. The degree of the agreement was evaluated by the weight kappa coefficient (κ). A slight agreement was assessed with a κ value ≤0.2, fair agreement for 0.21–0.4, moderate agreement for 0.41–0.6, substantial agreement for 0.61–0.8, and almost perfect agreement for >0.8 (27).

Furthermore, the Bland–Altman plot was used to illustrate the agreement between the DP or the HR and the WD when estimating the total intakes of energy and nutrient. A log-transformation of the values for the participants was performed to normalize the data (28). The mean of the two methods (DP with WD or HR with WD) in the *x*-axis was plotted against the differences between the two methods (DP–WD or HR–WD) in the *y*-axis, to examine whether the agreement between the methods varied with the energy and nutrients intakes. Pearson's correlation coefficient was calculated to test the association between the differences and the mean of the two methods. Additional zero bias lines (y = 0) and the limits of agreement (LOAs, calculated as mean ± 1.96 SD) were overlaid on the same plot. Ideally, the mean differences between the methods should be zero with no discernible bias, that is, the mean differences should cluster on the horizontal zero bias line. Any deviation of the mean differences line from the line of equality or any systematic variation of the differences in dietary intake across the range of dietary intake suggests the presence of additional systematic bias or limited agreement between the two methods (29, 30).

The estimates of the least detectable effect sizes (the least detectable degree to which the null hypothesis is indexed by the discrepancy between the null hypothesis and alternate hypothesis) specific to the study sample size and the types of analyses were calculated. A sample of 40 achieves 83% power to detect of a non-equivalent difference of 10%, with an α of 0.05 using a two-tailed test.

## Results

In this present study, 48 preschoolers were initially recruited. Of those, 8 children with metabolic diseases (*n* = 2), food-related allergy (*n* = 3), diarrhea or constipation (*n* = 3) were excluded. Finally, a total of 40 preschoolers were included, and 57.5% of them were girls. The participants were on average 4.9 (SD 1.0) years old, with a range from 4 to 6 years old. The median (Q1, Q3) BMI was 15.3 (14.5, 16.3) kg/m^2^. The socioeconomic characteristics, e.g., educational level of the parents and family income of these 40 participants are also shown in Supplementary Table 1.

The individual mean differences and correlations of energy and dietary nutrient intakes between the DP and the WD or between the HR and the WD are shown in Table 1. The intake of carbohydrate was significantly underestimated using the DP compared with using the WD, but the intake of energy and the other nutrients did not differ between the DP and the WD. As for the differences between the HR and the WD, the intakes of energy and nutrients were significantly lower in the HR except for the intakes of protein, total fat, vitamin E, sodium, and calcium, which were not different from these recorded in the WD. Additionally, the individual mean differences for energy and most nutrients between the DP and the WD were significantly lower than those between the HR and the WD, except for the total fat. For all dietary intake, the correlation coefficients between the DP and the WD ranged from 0.73 to 0.94, while 0.46 to 0.80 between the HR and the WD. The high correlation coefficients (≥0.70) were found for energy and all nutrients in the DP, as well as vitamin A, B2, C, potassium, calcium, and selenium in the HR. The Spearman's rank correlation coefficients (0.40–0.69) were moderate for energy and the remaining nutrients in the HR. Furthermore, the correlation coefficients between the DP and the HR (ranging from 0.40 to 0.83) were close to the coefficients between the HR and the WD, which to some extent indicated comparability between the DP and the WD. Absolute dietary intakes recorded in the WD, the DP, and the HR are also presented in Supplementary Table 2.

**Table 1:** The differences and associations of daily intakes of energy and nutrients recorded in the 24 h dietary recall (HR), diet photography record (DP), and weighed dietary record (WD) in the preschoolers from southwest China (*n* = 40).

**Nutrient intake**	**Individual mean differences (DP-WD)**		**Individual mean differences (HR-WD)**		**Individual mean differences (DP-HR)**	
	**Median (Q1, Q3)**	**Correlation**	**Median (Q1, Q3)**	**Correlation**	**Median (Q1, Q3)**	**Correlation**
Energy, Kcal	−94.64 (−257.40, 152.05)	0.85^##^	−179.01 (−515.60, 82.98)^*^	0.46^##^	117.28 (−113.68, 317.68)^*^	0.47^#^
Protein, g	−0.35 (−6.43, 13.28)	0.76^##^	−4.16 (−20.81, 6.50)	0.50^#^	8.76 (−4.90, 43.94)^*^	0.40
Total fat, g	−1.25 (−7.84, 11.82)	0.73^##^	−6.19 (−17.80, 10.35)	0.68^##^	1.61 (−5.89, 13.18)	0.68^##^
Carbohydrate, g	−12.38 (−38.42, 2.00)^*^	0.94^##^	−19.72 (−79.83, 6.49)^**^	0.56^##^	18.45 (−9.96, 43.94)^*^	0.54^##^
Fiber, g	−0.01 (−1.94, 0.85)	0.85^##^	−1.24 (−3.15, 0.17)^**^	0.62^##^	0.61 (−1.11, 2.38)	0.63^##^
Vitamin A, μg	14.19 (−46.07, 53.11)	0.94^##^	−24.01 (−117.10, 4.93) ^**^	0.72^##^	55.18 (7.33, 126.64)^**^	0.80^##^
Vitamin B1, mg	−0.02 (−0.17, 0.09)	0.82^##^	−0.12 (−0.40, 0.02) ^**^	0.60^##^	0.14 (−0.04, 0.33)^**^	0.48^#^
Vitamin B2, mg	−0.01 (−0.08, 0.15)	0.81^##^	−0.09 (−0.20, 0.04) ^*^	0.80^##^	0.11 (−0.06, 0.23)^*^	0.67^##^
Vitamin C, mg	−2.58 (−15.38, 14.90)	0.87^##^	−12.07 (−41.71, 3.39) ^*^	0.85^##^	10.93 (−7.84, 30.51)^*^	0.77^##^
Vitamin E, mg	−0.31 (−2.67, 5.33)	0.83^##^	−0.49 (−5.19, 1.77)	0.79^##^	1.18 (−0.96, 5.92)^*^	0.83^##^
Potassium, mg	7.61 (−155.34, 222.05)	0.82^##^	−149.67 (−444.30, 89.09) ^*^	0.73^##^	248.33 (−143.29, 661.45)^*^	0.70^##^
Sodium, mg	68.13 (−564.01, 739.41)	0.78^##^	−458.06 (−974.21, 496.87)	0.66^##^	377.54 (−270.87, 1129.99)^*^	0.54^##^
Calcium, mg	−7.69 (−39.93, 76.02)	0.77^##^	−37.16 (−86.02, 39.26)	0.78^##^	27.53 (−47.01, 92.59)^*^	0.68^##^
Magnesium, mg	−8.83 (−36.30, 22.50)	0.80^##^	−29.55 (−81.50, 10.39) ^*^	0.54^##^	21.84 (−8.69, 90.05)^*^	0.48^#^
Iron, mg	0.07 (−1.78, 2.39)	0.90^##^	−1.46 (−6.46, 1.00) ^*^	0.64^##^	2.44 (−1.36, 7.49)^*^	0.52^#^
Zinc, mg	−0.31 (−0.92, 1.46)	0.82^##^	−0.63 (−2.45, 0.44) ^*^	0.62^##^	0.37 (−1.01, 3.98)^*^	0.54^##^
Selenium, μg	0.77 (−6.59, 6.23)	0.92^##^	−2.49 (−12.98, 2.30) ^*^	0.79^##^	6.53 (−1.96, 13.85)^*^	0.73^##^

*^a^ For differences between DP and WD, HR and WD, or DP and HR were obtained by Wilcoxon's signed rank test*.

* *Significant at P < 0.05 level (two-tailed)*.

** *Significant at Bonferroni 0.0029 level (two-tailed)*.

# *Significant at P < 0.01 level (two-tailed) for correlation*.

## *Significant at Bonferroni 0.0006 level (two-tailed) for correlation*.

In total, 10 food groups (grains, legumes, vegetables, fruits, meat, poultry, sea foods, milk and dairy products, eggs, and snack foods) and 61 food items were assessed. The Spearman's rank correlation coefficients varied from 0.70 to 0.96 between the DP and the WD, while from 0.56 to 0.98 between the HR and the WD for different food groups. Except for sea foods, higher correlation coefficients with WD assessment were observed for the food groups recorded in the DP than in the HR. And the difference between the DP and WD were smaller than that of HR and WD for the intake of grains, legumes, vegetables, and sea foods (Supplementary Table 3).

Table 2 presents the potential misclassification of energy and nutrient intakes recorded in the DP or in the HR compared with those recorded in the WD. The proportion of preschoolers classified within the same or the adjacent quartile ranged from 90% for sodium and calcium, to 100% for carbohydrate, vitamin A, iron, and selenium in the DP in comparison with the WD. Classification into the same or the adjacent quartile was less than 95% for all the dietary nutrients in the HR, with the highest level for vitamin B2 (95.00%) and the lowest level for energy (72.50%). A substantial agreement (κ = 0.61–0.80) in ranking preschoolers according to their intake between the DP and the WD was observed for energy and 10 nutrients (total fat, carbohydrate, fiber, vitamin A, B2, C, potassium, magnesium, iron, and selenium). A moderate agreement (κ = 0.41–0.60) was seen for the other six nutrients (protein, vitamin B1, E, sodium, calcium, and zinc). However, the level of agreement between the HR and the WD varied from acceptable (κ = 0.21–0.40) for energy and for four nutrients (protein, carbohydrate, vitamin B1, and magnesium), to moderate for 11 nutrients (total fat, fiber, vitamin A, C, E, potassium, sodium, magnesium, iron, zinc, and selenium), to relatively high for other two nutrients (vitamin B2 and calcium). Except for sodium and calcium, greater degree of agreements with the WD between intake of all dietary nutrient were detected in the DP.

**Table 2:** Cross-classification for agreement among the daily intakes of energy and nutrients recorded in 24 h dietary recall (HR), diet photograph record (DP), and weighed dietary record (WD) in the preschoolers from Southwest China (*n* = 40).

	**Agreement for quartiles between DP and WD**					**Agreement for quartiles between HR and WD**				
	**Same/adjacent^a^**		**Opposite^b^**			**Same/adjacent^a^**		**Opposite^b^**		
**Nutrient intake**	***n***	**%**	***n***	**%**	**Weighted Kappa^c^**	***n***	**%**	***n***	**%**	**Weighted Kappa^c^**
Energy, Kcal	39	97.50	1	2.50	0.76 (0.62, 0.90)^**^	29	72.50	11	27.50	0.28 (0.04, 0.52)^*^
Protein, g	37	92.50	3	7.50	0.60 (0.43, 0.77)^**^	32	80.00	8	20.00	0.32 (0.11, 0.53)^**^
Total fat, g	38	95.00	2	5.00	0.62 (0.44, 0.81)^**^	35	87.50	5	12.50	0.47 (0.28, 0.65)^**^
Carbohydrate, g	40	100.00	0	0.00	0.80 (0.69, 0.92)^**^	35	87.50	5	12.50	0.40 (0.20, 0.60)^**^
Fiber, g	39	97.50	1	2.50	0.64 (0.49, 0.79)^**^	34	85.00	6	15.00	0.52 (0.31, 0.73)^**^
Vitamin A, μg	40	100.00	0	0.00	0.76 (0.64, 0.88)^**^	36	90.00	4	10.00	0.52 (0.33, 0.72)^**^
Vitamin B1, mg	38	95.00	2	5.00	0.52 (0.35, 0.69)^**^	32	80.00	8	20.00	0.37 (0.15, 0.59)^**^
Vitamin B2, mg	37	92.50	3	7.50	0.65 (0.48, 0.83)^**^	38	95.00	2	5.00	0.62 (0.45, 0.79)^**^
Vitamin C, mg	39	97.50	1	2.50	0.72 (0.58, 0.87)^**^	36	90.00	4	10.00	0.60 (0.42, 0.78)^**^
Vitamin E, mg	38	95.00	2	5.00	0.60 (0.44, 0.76)^**^	36	90.00	4	10.00	0.56 (0.38, 0.74)^**^
Potassium, mg	38	95.00	2	5.00	0.68 (0.52, 0.84)^**^	35	87.50	5	12.50	0.48 (0.28, 0.68)^**^
Sodium, mg	36	90.00	4	10.00	0.52 (0.34, 0.70)^**^	34	85.00	6	15.00	0.52 (0.32, 0.72)^**^
Calcium, mg	36	90.00	4	10.00	0.48 (0.30, 0.66)^**^	37	92.50	3	7.50	0.64 (0.46, 0.82)^**^
Magnesium, mg	37	92.50	3	7.50	0.64 (0.47, 0.81)^**^	34	85.00	6	15.00	0.40 (0.19, 0.61)^**^
Iron, mg	40	100.00	0	0.00	0.72 (0.59, 0.85)^**^	34	85.00	6	15.00	0.48 (0.29, 0.68)^**^
Zinc, mg	38	95.00	2	5.00	0.60 (0.44, 0.76)^**^	35	87.50	5	12.50	0.44 (0.25, 0.63)^**^
Selenium, μg	40	100.00	0	0.00	0.66 (0.53, 0.80)^**^	37	92.50	3	7.50	0.52 (0.34, 0.70)^**^

a *Same quartile—subjects classified into the same fourth; ‘Adjacent’ quartile—subjects differing by one category*.

b *Opposite quartile—subjects differing by two categories*.

c *The values are weighed kappa and their 95% CIs*.

* *Significant at P < 0.05 level (two-tailed)*.

** *Significant at Bonferroni 0.0029 level (two-tailed)*.

In addition, the Bland–Altman plots were considered for agreements in the energy and nutrients intakes between the DP or the HR and the WD. The individual differences in the energy and 11 nutrients (protein, total fat, vitamin A, B2, C, E, potassium, magnesium, calcium, iron, and zinc) between the DP and the WD or between the HR and the WD were not significantly associated with the means from using the three methods (Pearson's correlation coefficient: −0.32 to 0.18, *P* ≥ 0.05), which indicated that the variability and direction of the difference did not depend on the intake level. Except for carbohydrate intake, the geometric mean for energy and for the remaining 14 nutrients estimated by the DP was comparable with those with the WD, with a range of 2% above to 12% below. But only the geometric mean for five nutrients (total fat, vitamin B2, E, sodium, and calcium) recorded in the HR was comparable with the WD, with a range of 2% above to 8% below. On an average, the DP underestimated the intake of carbohydrate by 7%, compared with the WD. Furthermore, LOAs indicated that the DP could estimate carbohydrate intake within a range of 20% above to 28% below for most of the participants. The geometric mean difference for the intake of energy and 10 nutrients (carbohydrate, protein, vitamin A, B1, C, potassium, magnesium, iron, zinc, and selenium) between the HR and the WD showed that the HR underestimated the intake of these nutrients from 9 to 24% compared with the WD (Supplementary Table 4).

**Figure 3:**
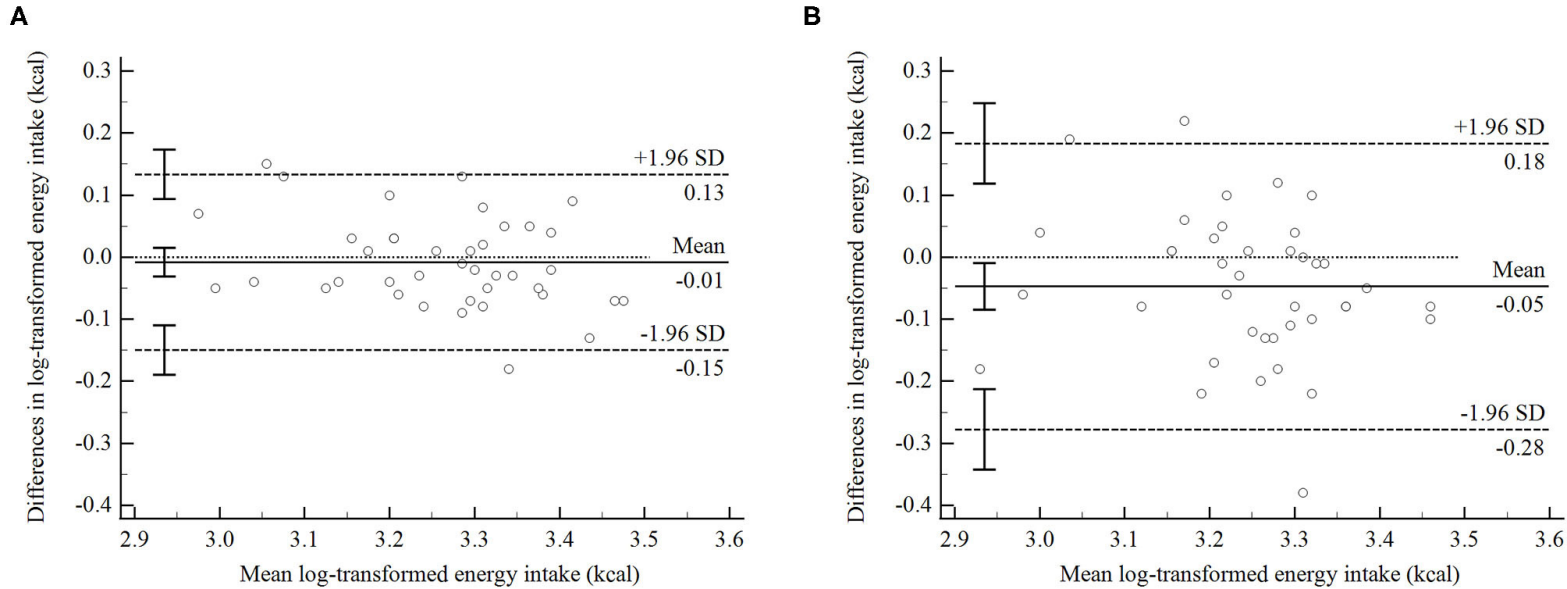
The Bland–Altman plots of agreement between energy intake recorded in 24-h dietary recall (HR) and weighed dietary record (WD), or between diet photography record (DP) and weighed dietary record (WD) in Chinese preschoolers (*n* = 40). Data are log-transformed values. The difference between energy intake calculated from the DP and the WD or from the HR and the WD for each participant (*y*-axis) is plotted against the mean energy or nutrients intake averaged from above three methods (*x*-axis). **(A)** The agreement between the DP and the WD, while the **(B)** shows the agreement between the HR and the WD.

Figure 3 and Supplementary Figure 1 present the Bland–Altman plots for energy and six important nutrients for preschoolers (carbohydrate, protein, fat, vitamin A, calcium, and iron) between the DP or the HR and the WD as examples. On average, the energy intake was underestimated by 2%, while protein, fat, vitamin A, Fe, and calcium intakes were overestimated in the DP by 5, 5, 7, 2 and 5%, respectively, compared with the WD. For most of the participants, the DP estimated energy and the above nutrients intake within a range of 104% above to 42% below these recorded in the WD. Moreover, compared with the HR, the average differences between energy and these six nutrients obtained by the DP and the WD were closer to 0, and the data distributions were more concentrated.

## Discussion

The current study indicates the relative validity of the DP for measuring energy and nutrient of daily meals consumed by the preschoolers in the kindergarten setting. Although there was a tendency for underestimating dietary intake for both the methods, the smaller differences from known weights and the better agreements between estimates with the WD were obtained by the DP over the HR.

The correlation coefficients are useful for determining whether there is a linear trend in the response between the test and reference method. Except for intake of energy recorded in the HR, the correlation coefficients for dietary intake recorded in both the DP and the HR were higher than 0.5, which has been proposed to indicate the validity (31). In addition, the coefficients for dietary intake in the DP were generally higher than that in the HR. Meanwhile, weighted κ and the Bland–Altman statistics were also employed to ascertain validity. The weighted κ statistic should be >0.4 to confirm at least a moderate agreement (32, 33). In our study, the weighted κ values for energy and nutrient intakes from the DP were all >0.4, while the weighted κ values for energy, protein, carbohydrate, vitamin B1, and magnesium intakes from the HR were smaller than 0.4. The proportions of preschoolers who were correctly classified were >90%, and the misclassification rate was 10% or less for energy and nutrient intakes using the DP. The Bland–Altman plots which were produced as a popular comparative tool for agreement research (34), suggested good agreement for the dietary intake estimated using the DP and the WD, indicating that the DP and the WD were used for estimating daily dietary intake of the preschoolers were interchangeable to some extent. As evidenced from the correlation coefficients, cross-classifications, and Bland-Altman plots, the DP and the HR had moderate to good validity for estimating energy and nutrient among the preschoolers, and the DP showed a higher level of validity over the HR.

In the current study, the DP was found to underestimate the energy intake by only 2%, which is similar to the reported 3.7 or 4% in an American adult and is smaller than the reported 7.5% among the African American preschoolers (19, 24, 35). While the degree of error for the HR was higher (−21 to 43%) when compared with the DP, and the error was close to the other self-reported methods with an error of 37% or more (36, 37). The previous studies show that the error of self-reported methods are partly due to inaccurate estimation of portion size by the participants (38). The DP does not rely on the participants to estimate portion sizes, which is likely one reason for its better accuracy than HR. Although the DP and the HR underestimated most of nutrient intakes of the preschoolers, mean errors were relatively smaller for intake recorded in the DP, and the agreement tests found that estimated intake in grams from the DP was more equivalent to the weighted intake compared with the HR. Thus, the DP appears to be a promising method for estimating dietary intake of the preschoolers.

However, the DP used in our study significantly underestimated the intake of snacks and grains compared with the WD. This inaccuracy on snacks may reflect the small quantities of snacks frequently consumed by the preschoolers, which can easily lead to underreporting and underestimation (18, 39). Foods in the grain category, such as rice, black rice, whole millet, corn, noodle, rice porridge, and steamed bread. Similar results of the underestimated grains were observed among the adult (40) and children (41) using the DP. But the source of these underestimations is still unclear. In general, the DP yielded closer estimates to the actual weight of most food groups over the HR.

Given the importance of estimating the dietary intake of preschooler for intervention and research, the DP have significant advantages. First, the DP is a promising tool to obtain accurate dietary estimates from the free-living individuals, without increasing burden to the participants. Second, the food images sent to the server provide a possibility for quick feedback about the missing data or incomplete data to increase the report accuracy. Finally, public health and epidemiological research on a large scale could be conducted with the help of the DP for collecting and analyzing the dietary data remotely.

Nevertheless, our experience during this study reminds us that some barriers need to be modified in the DP. To reduce the missing images is the first difficulty to overcome. There were no missing data in our study by supervising and reminding the parents in the kindergarten. Automated prompts reminder during the meals should be installed into the app when the DP are applied to home settings. The estimates of the DP rely on the food images with known weight, thus establishing a food photograph database covering more food groups with different cooking methods is another challenge. We found that updating of the pictures for small quantities of foods is an urgent need. Finally, the food identification and estimation by the human raters need more than 30 min for a sample, and the development of computer imaging algorithms is a further goal to enhance the efficiency of the DP.

The present study has several important strengths. It was the first study to assess the total daily intake of the preschoolers in China using the DP and evaluate the relative validity compared with the WD. Moreover, the preschoolers were given a large number of food choices within the food groups and should have been able to satisfy their preferences, allowing the diet to be as normal as possible. Additionally, the DP, a relatively new dietary method, and the HR, a traditional dietary method were tested in the same sample, which allows direct comparisons of the accuracy between these two methods. A further advantage lies in the use of different statistical methods to determine agreement.

Some limitations should be considered. First, the study has a relatively small sample size. But our sample size of 40 preschoolers is acceptable compared with the other validation studies in preschool children (22–24, 42). The study was conducted in a controlled kindergarten setting, and the validity of the DP needs to be test in a free-living condition. Furthermore, we did not allow the children to share snacks as is commonly done in China, altering the normal eating behavior. However, we covered different normal diet to simulate the naturalistic setting, and the preschoolers were permitted to choose what they like in the three regular meals, which suggests the potential use of the DP in future research. For the application of the HR method, we did not perform the multiple pass method, which may have led to some underestimation of energy intake, since conducting multiple passes is shown to increase the reported energy intake over not performing multiple passes (43). To ensure better accuracy of the dietary assessment, dietary recall using the multiple pass method is recommended for use in future studies in which dietary recall is conducted. Finally, we only studied 1 day of dietary intake. Given substantial variability in dietary intake, investigation on the validity of the DP for evaluating the habitual intake are needed.

In conclusion, the DP is a valid assessment instrument for measuring the energy and nutrient intake and is more suitable than the HR among the preschoolers.

## Data Availability Statement

The raw data supporting the conclusions of this article will be made available by the authors, without undue reservation.

## Ethics Statement

The studies involving human participants were reviewed and approved by Ethics Committee of Sichuan University. Written informed consent to participate in this study was provided by the participants' legal guardian/next of kin.

## Author Contributions

YJX, RND, PF, WKG, and DX collected the data. YJX conducted the analysis and drafted the manuscript under the guidance and supervision of GC. All the authors read and reviewed the manuscript and approved the final manuscript.

## Funding

The study was supported by Study of Diet and Nutrition Assessment and Intervention Technology (No. 2020YFC2006300) from Active Health and Aging Technologic Solutions Major Project of National Key R&D Program.

## Conflict of Interest

The authors declare that the research was conducted in the absence of any commercial or financial relationships that could be construed as a potential conflict of interest.

## Publisher's Note

All claims expressed in this article are solely those of the authors and do not necessarily represent those of their affiliated organizations, or those of the publisher, the editors and the reviewers. Any product that may be evaluated in this article, or claim that may be made by its manufacturer, is not guaranteed or endorsed by the publisher.
