# Supplementary material for: Comparison of the Diet Photograph Record to Weighed Dietary Record and 24 h Dietary Recall for Estimating Energy and Nutrient Intakes Among Chinese Preschoolers
Source: Front Nutr. 2021 Nov 11;8:755683. doi: 10.3389/fnut.2021.755683 (PMC8631866; doi:10.3389/fnut.2021.755683)
Supplement: Supplementary file 1 [file Table_1.DOCX]

**Supplementary Table 1 General characteristics of the participants in the present study^1^ (n = 40).**

| **Characteristics** | **Values** |
| --- | --- |
| Female (n (%)) | 23 (57.50) |
| Age (years) | 4.9 ± 1.0 |
| Anthropometry |  |
| Height (m) | 109.0 (104.0, 115.0) |
| Weight (kg) | 18.8 (16.5, 19.8) |
| BMI (kg/m^2^) | 15.3 (14.5, 16.3) |
| Education level of father^2^ (n (%)) | 38 (95.0) |
| Education level of mother^2^(n (%)) | 35 (87.5) |
| Family income^3^ (n (%)) | 38 (95.0) |

^1^Data are presented as median (Q1, Q3), Mean ±SD, or percentage.

^2^At least 16 years of school education.

^3^The family average annual income more than 60,000 CNY(Chinese yuan), which indicates a middle income level in China according to National Bureau of Statistics reported in 2017 (1).

**References**

(1) China, N.B.o.S.o., *China Statistical Yearbook*. 2017: China Statistics Press.
