# Supplementary material for: Comparison of the Diet Photograph Record to Weighed Dietary Record and 24 h Dietary Recall for Estimating Energy and Nutrient Intakes Among Chinese Preschoolers
Source: Front Nutr. 2021 Nov 11;8:755683. doi: 10.3389/fnut.2021.755683 (PMC8631866; doi:10.3389/fnut.2021.755683)
Supplement: Supplementary file 2 [file Table_2.DOCX]

**Supplementary Table 2. Nutrient intakes** **reported in the 24h dietary recall (HR), diet photography record (DP) and weighed dietary record (WD) in preschoolers from southwest China (n=40).**

| Nutrient intake | WD | DP | HR |
| --- | --- | --- | --- |
| Energy, Kcal | 1948.6(1503.7, 2285.5) | 1842.9(1505.9,2217.3) | 1660.8(1445.4, 2058.9) |
| Protein, g | 71.8(56.4, 90.0) | 77.4(63.0, 90.9) | 66.3(55.7, 82.0) |
| Total fat, g | 63.2(47.8, 88.5) | 65.6(53.3, 88.4) | 66.5(47.7, 85.7) |
| Carbohydrate, g | 236.8(188.0,298.8) | 202.1(187.1, 279.5) | 202.1(168.2, 248.1) |
| Fiber, g | 11.6(8.5, 14.1) | 11.1(8.1, 13.9) | 9.4(7.0, 12.6) |
| Vitamin A, μg | 263.0(165.1, 494.7) | 319.6(190.4, 436.9) | 216.2(120.6, 308.9) |
| Vitamin B1, mg | 0.9(0.7, 1.2) | 0.8(0.7, 1.1) | 0.7(0.6, 1.0) |
| Vitamin B2, mg | 1.0(0.8, 1.2) | 1.0(0.8, 1.2) | 0.9(0.8, 1.0) |
| Vitamin C, mg | 93.8(43.0, 129.3) | 85.7(44.3, 115.2) | 64.9(28.5, 99.7) |
| Vitamin E, mg | 20.2(8.8, 28.2) | 23.4(10.9, 27.7) | 17.3(7.5, 27.7) |
| Potassium, mg | 1906.4(1511.9, 2429.1) | 1972.2(1514.5, 2407.7) | 1636.4(1308.1, 2085.0) |
| Sodium, mg | 3758.4(2636.7, 4436.8) | 4054.0(3261.9, 4915.0) | 3795.7(2636.7, 4436.8) |
| Calcium, mg | 498.3(356.3, 598.4) | 519.8(399.9, 710.4) | 470.1(361.9, 585.8) |
| Magnesium, mg | 273.8(205.7, 356.3) | 280.6(205.6, 316.5) | 218.9(189.4, 294.1) |
| Iron, mg | 17.9(12.3, 23.3) | 19.7(13.0, 23.6) | 16.3(12.6, 19.9) |
| Zinc, mg | 10.7(7.3, 13.0) | 10.3(8.0, 13.2) | 8.7(7.7, 11.4) |
| Selenium, μg | 42.1(26.7, 62.2) | 44.6(33.0, 57.0) | 38.0(25.8, 49.2) |

*Data are presented as median (Q1, Q3).
