# Supplementary material for: Comparison of the Diet Photograph Record to Weighed Dietary Record and 24 h Dietary Recall for Estimating Energy and Nutrient Intakes Among Chinese Preschoolers
Source: Front Nutr. 2021 Nov 11;8:755683. doi: 10.3389/fnut.2021.755683 (PMC8631866; doi:10.3389/fnut.2021.755683)
Supplement: Supplementary file 3 [file Table_3.DOCX]

**Supplementary Table 3. Differences and associations of daily food intakes reported in the diet photography record (DP), 24h dietary recall (HR) and weighed dietary record (WD) in preschoolers from southwest China (n=40).**

|  | **Individual Mean Differences**  **(DP-WD)** | | |  | **Individual Mean Differences**  **(HR-WD)** | |  | **Individual Mean Differences**  **(DP-HR)** | |
| --- | --- | --- | --- | --- | --- | --- | --- | --- | --- |
| **Nutrient intake** | **Median (Q1, Q3)** | **Correlation** | |  | **Median (Q1, Q3)** | **Correlation** |  | **Median (Q1, Q3)** | **Correlation** |
| Grains | -6.0 (-27.0, 5.6)^**^ | | 0.96^##^ |  | -10.0 (-47.0, 21.3)^**^ | 0.81^##^ |  | 0.0 (-10.0, 35.5)^*^ | 0.85^##^ |
| Legumes | 2.6 (-0.5, 10.0)^*^ | | 0.94^##^ |  | -2.1 (-21, 1.3) | 0.62^#^ |  | 3.1 (-1.1, 38.8)^*^ | 0.61^#^ |
| Vegetables | -0.3 (-8.0, 4.0)^*^ | | 0.95^##^ |  | -7.0 (-28.5, 5.8)^**^ | 0.75^##^ |  | 5.0 (-5.0, 20.0)^*^ | 0.75^##^ |
| Fruits | 6.0 (-4.0, 23.0) | | 0.93^##^ |  | 17.0 (-10.0, 36.0) | 0.59^#^ |  | 0.0 (-20.0, 17.5) | 0.58^#^ |
| Meat | 1.3 (-11.1, 13.0) | | 0.74^##^ |  | -2.0 (-13.4, 4.3) | 0.71^##^ |  | 5.0 (-7.5, 30.0) | 0.65^##^ |
| Poultry | 8.0 (-8.5, 27.5) | | 0.74^##^ |  | 1.1 (-32.0, 30.4) | 0.57^#^ |  | 3.9 (-27.9, 48.9) | 0.51^#^ |
| Milk | 0.0 (0.0, 0.0) | | 0.87^##^ |  | 0.0 (0.0, 0.0) | 0.57^#^ |  | 0.0 (0.0, 0.0) | 0.46 |
| Eggs | 5.0 (1.0, 11.0)^*^ | | 0.70^##^ |  | 5.5 (-1.1, 11.0)^*^ | 0.56^#^ |  | 0.0 (0.0, 0.0) | 0.60^##^ |
| Sea foods | -2.5 (-5.5, 2.5) | | 0.90^#^ |  | 27.5 (2.5, 59.6)^*^ | 0.98^##^ |  | -40.0 (-56.2, -7.5)^*^ | 0.95^##^ |
| Snack foods | -1.0 (-8.0, -0.5)^**^ | | 0.91^##^ |  | 0.0 (-11.2, 20.4) | 0.78^##^ |  | -1.0 (-20.0, 5.0) | 0.83^##^ |

^a^ For differences between DP and WD, HR and WD, or DP and HR were obtained by Wilcoxon signed rank test.

^*^Significant at P＜0.05 level (two-tailed).

^**^Significant at Bonferroni 0.005 level (two-tailed).

^#^Significant at P＜0.01 level (two-tailed) for correlation.

^##^Significant at Bonferroni 0.001 level (two-tailed) for correlation.
