# Supplementary material for: Comparison of the Diet Photograph Record to Weighed Dietary Record and 24 h Dietary Recall for Estimating Energy and Nutrient Intakes Among Chinese Preschoolers
Source: Front Nutr. 2021 Nov 11;8:755683. doi: 10.3389/fnut.2021.755683 (PMC8631866; doi:10.3389/fnut.2021.755683)
Supplement: Supplementary file 4 [file Table_4.DOCX]

**Supplementary Table 4. Values of Bland-Altman plots for agreement between daily intakes of energy and nutrients reported in the diet photography record (DP), 24h dietary recall (HR) and weighed dietary record (WD) in preschoolers from southwest China (n=40).**

|  | **Geometric differences**  **(DP vs WD)** | |  | **Limits of agreement ^a^**  **(DP vs WD)** | |  |  | **Geometric differences**  **(HR vs WD)** | |  | **Limits of agreement ^a^**  **(HR vs WD)** | |  |  |
| --- | --- | --- | --- | --- | --- | --- | --- | --- | --- | --- | --- | --- | --- | --- |
| **Nutrient intake** | **Mean** | **SD** |  | **Low** | **Upper** | **R^b^** | ***P*** | **Mean** | **SD** |  | **Low** | **Upper** | **R^b^** | ***P*** |
| Energy, Kcal | 0.98 | 1.17 |  | 0.71 | 1.35 | -0.18 | 0.052 | 0.89 | 1.32 |  | 0.52 | 1.51 | -0.20 | 0.225 |
| Protein, g | 1.05 | 1.20 |  | 0.72 | 1.55 | -0.08 | 0.497 | 0.91 | 1.32 |  | 0.54 | 1.58 | 0.11 | 0.528 |
| Total fat, g | 1.05 | 1.32 |  | 0.58 | 1.86 | -0.13 | 0.373 | 0.98 | 1.32 |  | 0.55 | 1.74 | -0.09 | 0.515 |
| Carbohydrate, g | 0.93 | 1.12 |  | 0.72 | 1.20 | -0.15 | 0.006 | 0.85 | 1.38 |  | 0.46 | 1.62 | -0.31 | 0.065 |
| Vitamin A, μg | 1.07 | 1.38 |  | 0.58 | 2.04 | -0.27 | 0.061 | 0.76 | 1.78 |  | 0.23 | 2.40 | -0.14 | 0.219 |
| Vitamin B1, mg | 1.00 | 1.26 |  | 0.60 | 1.62 | -0.24 | 0.018 | 0.79 | 1.38 |  | 0.43 | 1.51 | -0.15 | 0.253 |
| Vitamin B2, mg | 1.02 | 1.20 |  | 0.71 | 1.51 | -0.13 | 0.129 | 0.93 | 1.23 |  | 0.60 | 1.45 | -0.08 | 0.456 |
| Vitamin C, mg | 1.00 | 1.91 |  | 0.28 | 3.55 | -0.01 | 0.971 | 0.76 | 1.91 |  | 0.21 | 2.69 | 0.09 | 0.356 |
| Vitamin E, mg | 1.10 | 1.45 |  | 0.51 | 2.29 | -0.06 | 0.544 | 0.93 | 1.51 |  | 0.42 | 2.14 | 0.04 | 0.701 |
| Potassium, mg | 1.02 | 1.17 |  | 0.72 | 1.45 | -0.19 | 0.059 | 0.89 | 1.23 |  | 0.58 | 1.38 | 0.14 | 0.234 |
| Sodium, mg | 1.12 | 1.58 |  | 0.44 | 2.88 | -0.65 | 0.001 | 0.98 | 1.78 |  | 0.32 | 3.02 | -0.40 | 0.039 |
| Calcium, mg | 1.05 | 1.29 |  | 0.65 | 1.74 | 0.01 | 0.912 | 0.93 | 1.35 |  | 0.52 | 1.70 | 0.12 | 0.352 |
| Magnesium, mg | 0.98 | 1.20 |  | 0.65 | 1.45 | -0.19 | 0.094 | 0.83 | 1.41 |  | 0.41 | 1.66 | 0.04 | 0.835 |
| Iron, mg | 1.02 | 1.20 |  | 0.69 | 1.51 | -0.07 | 0.390 | 0.87 | 1.35 |  | 0.46 | 1.62 | -0.22 | 0.125 |
| Zinc, mg | 1.02 | 1.23 |  | 0.71 | 1.51 | -0.03 | 0.756 | 0.89 | 1.29 |  | 0.54 | 1.51 | -0.07 | 0.612 |
| Selenium, μg | 1.07 | 1.26 |  | 0.68 | 1.70 | -0.24 | 0.001 | 0.89 | 1.35 |  | 0.49 | 1.62 | -0.11 | 0.275 |

^a^ The limits of agreement were calculated by the mean difference plus or minus 1.96 standard deviations (d ± 1.96 SD).

^b^ Slopes of fitted regression lines.
