# Supplementary material for: Comparison of the Diet Photograph Record to Weighed Dietary Record and 24 h Dietary Recall for Estimating Energy and Nutrient Intakes Among Chinese Preschoolers
Source: Front Nutr. 2021 Nov 11;8:755683. doi: 10.3389/fnut.2021.755683 (PMC8631866; doi:10.3389/fnut.2021.755683)
Supplement: Supplementary file 5 [file Data_Sheet_1.PDF]

Supplementary Figure 1. Bland-Altman plots of agreement between nutrients intake reported in 24-h dietary recall (HR) and weighed dietary record (WD), or between diet photography record (DP) and weighed dietary record (WD) in Chinese preschoolers (n=40). The left parts of the (A)~(F) present the agreement between the DP and the WD for intake of carbohydrate, protein, fat, vitamin A, calcium and iron, respectively, while the right parts show the agreement of the same nutrients between the HR and the WD.

(A)

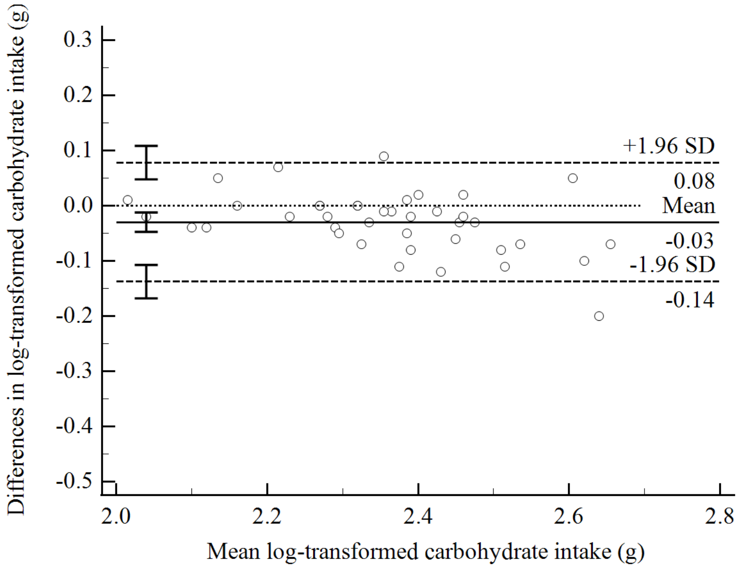

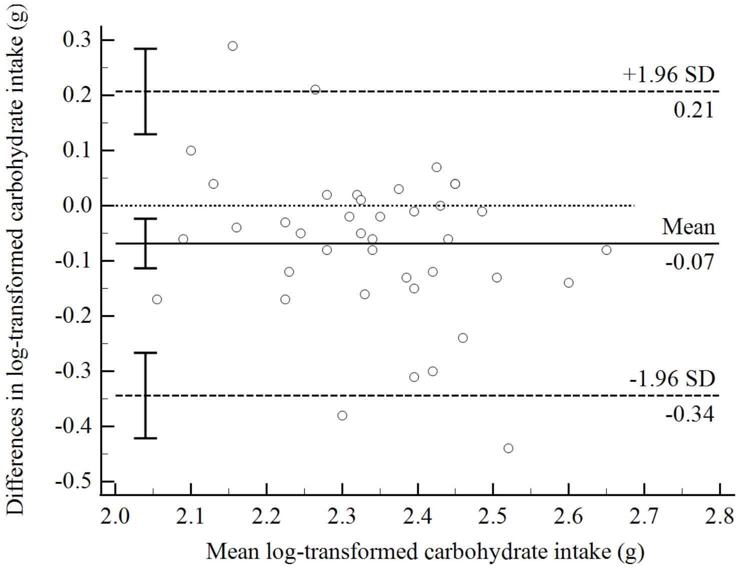

(B)

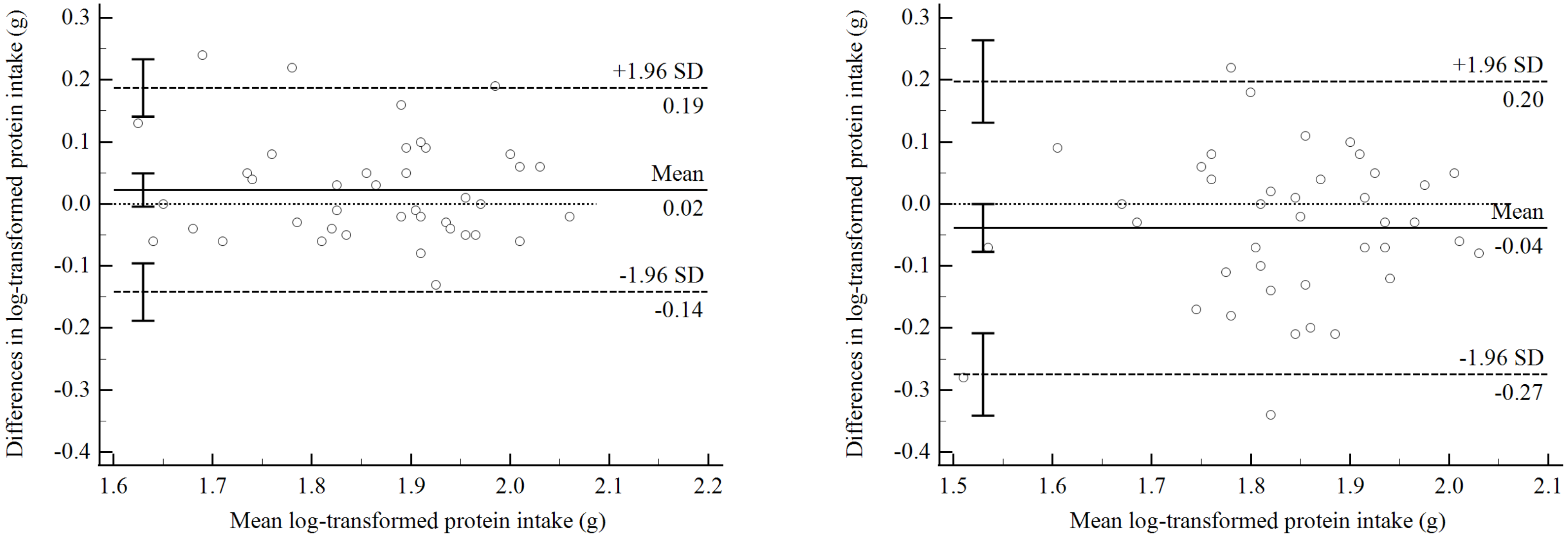

Differences in log-transformed protein intake (g)
Mean log-transformed protein intake (g)
+1.96 SD
0.19
Mean
0.02
-1.96 SD
-0.14
Differences in log-transformed protein intake (g)
Mean log-transformed protein intake (g)
+1.96 SD
0.20
Mean
-0.04
-1.96 SD
-0.27

(C)

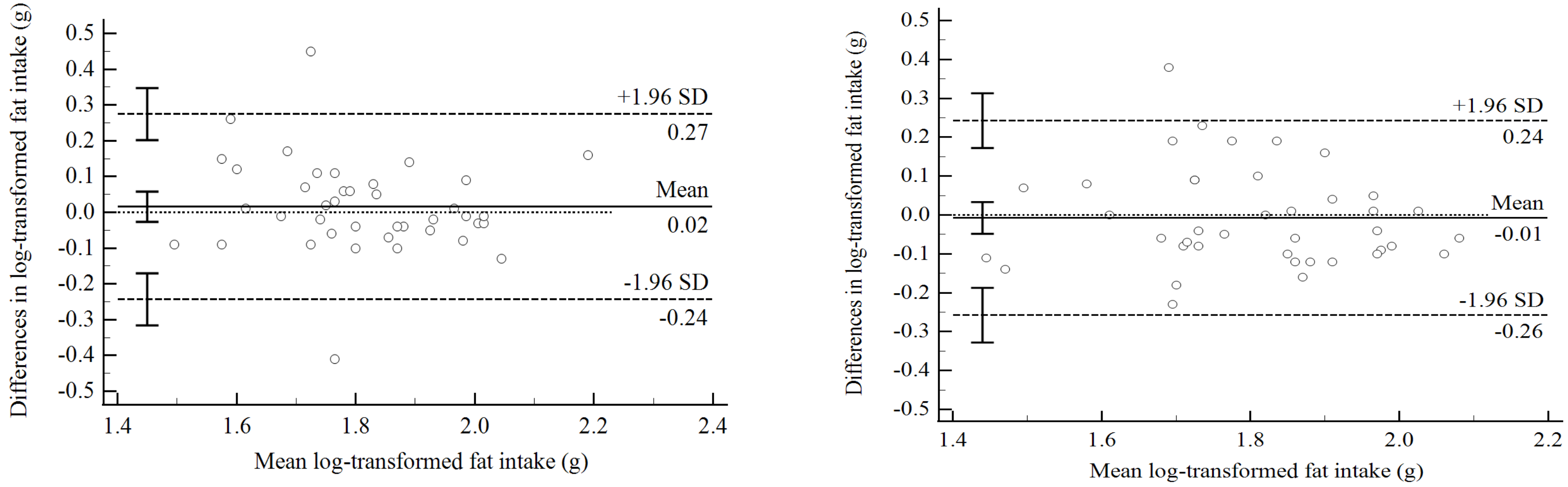

Differences in log-transformed fat intake (g)
Mean log-transformed fat intake (g)
+1.96 SD
0.27
Mean
0.02
-1.96 SD
-0.24
+1.96 SD
0.24
Mean
-0.01
-1.96 SD
-0.26

(D)

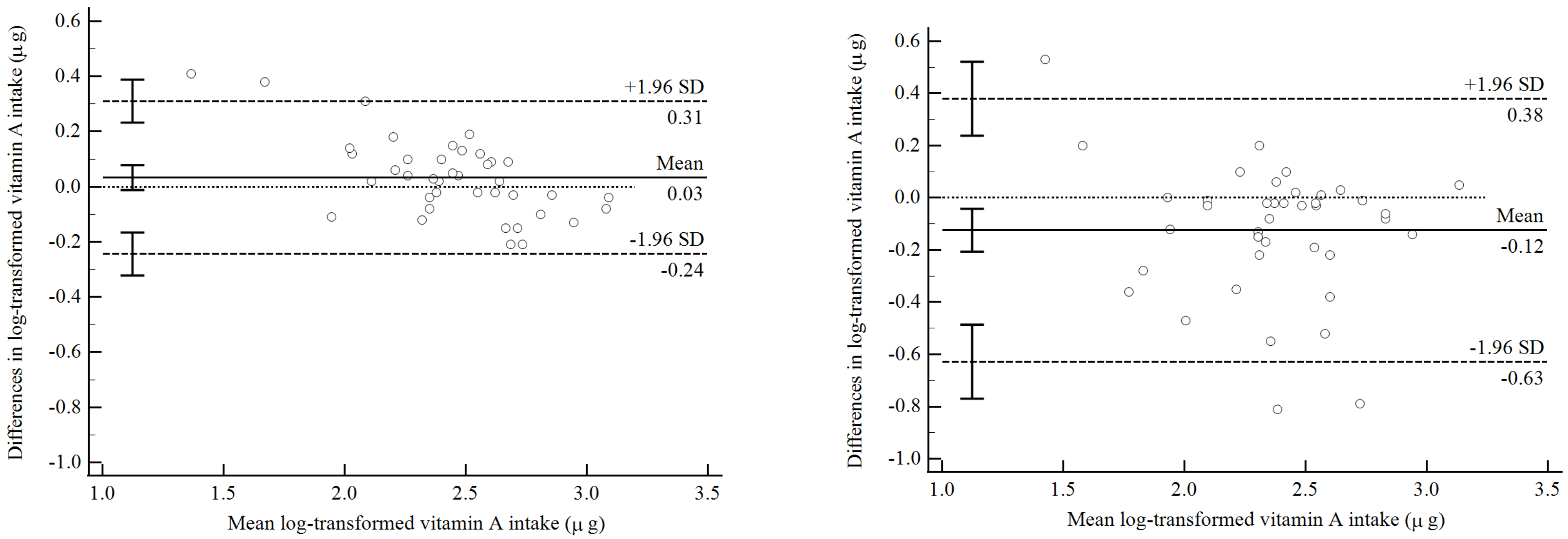
Differences in log-transformed vitamin A intake (μ g)
Mean log-transformed vitamin A intake (μ g)
+1.96 SD
0.31
Mean
0.03
-1.96 SD
-0.24
+1.96 SD
0.38
Mean
-0.12
-1.96 SD
-0.63

(E)

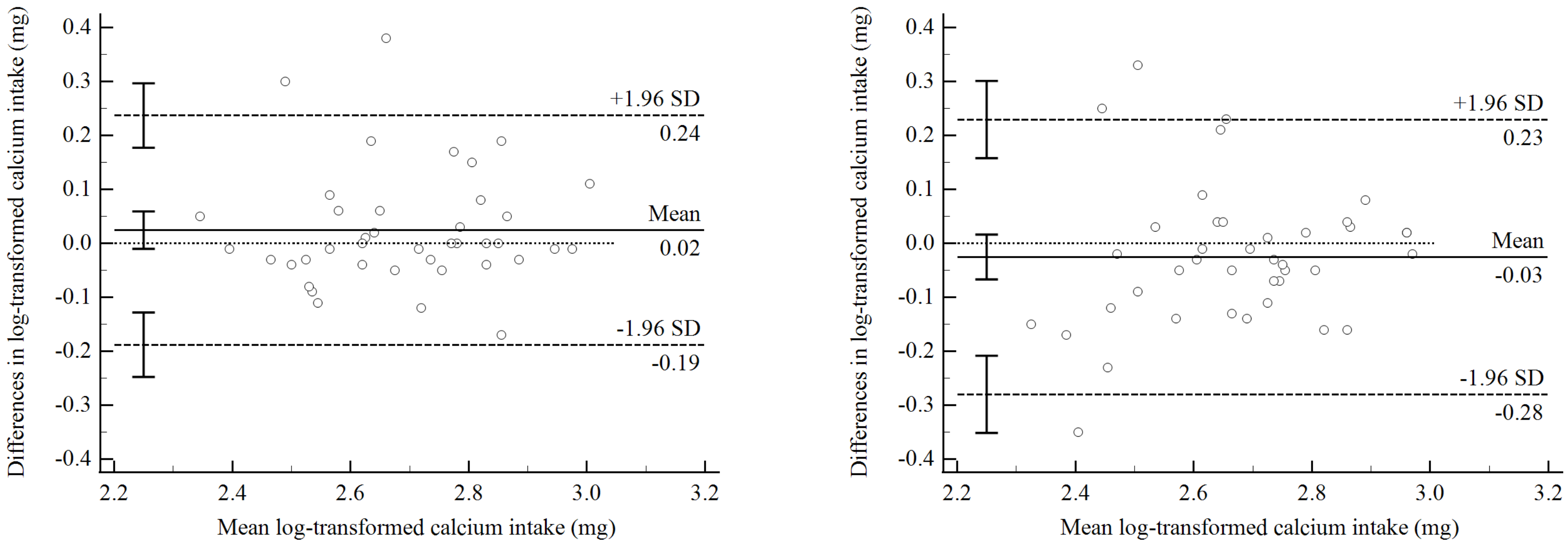
Differences in log-transformed calcium intake (mg)
0.4
0.3
0.2
0.1
0.0
-0.1
-0.2
-0.3
-0.4
+1.96 SD
0.24
Mean
0.02
-1.96 SD
-0.19
2.2
2.4
2.6
2.8
3.0
3.2
Mean log-transformed calcium intake (mg)
Differences in log-transformed calcium intake (mg)
+1.96 SD
0.23
Mean
-0.03
-1.96 SD
-0.28
Mean log-transformed calcium intake (mg)

(F)

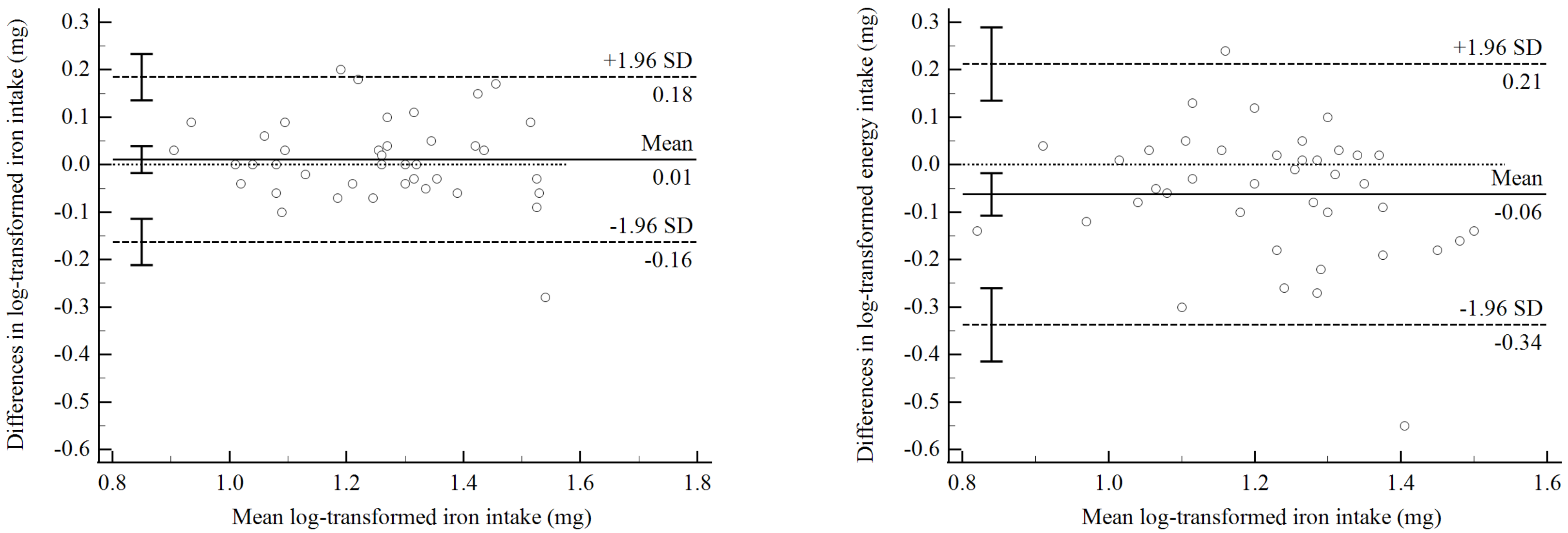

+1.96 SD
0.18
Mean
0.01
-1.96 SD
-0.16
Differences in log-transformed iron intake (mg)
Mean log-transformed iron intake (mg)
+1.96 SD
0.21
Mean
-0.06
-1.96 SD
-0.34
Differences in log-transformed energy intake (mg)
Mean log-transformed iron intake (mg)
